# Editorial: Current methods for detecting and diagnosing stem cell pathogenesis: a focus on translational interventions in the contemporary disease and technology landscape

**DOI:** 10.3389/fcell.2025.1558476

**Published:** 2025-02-24

**Authors:** Prasad S. Koka, Birgitta Sundell-Ranby

**Affiliations:** ^1^ Biomedical Research Institute of Southern California, Oceanside, CA, United States; ^2^ Wayne State University, Detroit, MI, United States

**Keywords:** disease diagnosis and analysis, stem cell therapies, epigenetic regulation, stem cell differentiation potential, noncoding RNA (ncRNA) regulation, wound healing, spinal cord injury, regenerative medicine

Understanding the mechanisms of disease pathogeneses wherein stem cells of different differentiation potencies are involved, can lead to the development of suitable efficacious molecular or cellular therapies. One such example of realizing the molecular mechanisms to develop translational interventions are the mechanisms and therapies for clinical diagnosis of the chronic hematological disorders (cytopenias) in HIV/AIDS by using quantitative RT-PCR of HOXB3 homeobox transcription factor messenger RNA (Koka and Ramdass, 2024; Padmanabhan et al., 2020). Further, as in this regard, such techniques potentially carry wider extended and improved diagnostic applications to the detection and treatments of hematological malignancies (leukemias), with potential involvement of microRNA candidates in relevance to both the occurrence and control of the disease.

In an article of this Research Topic collection, therapeutic considerations to potentially resolve the persistence of HIV latency not only in the mature differentiated, but also in early stage or primitive progenitor hematopoietic stem cells, are presented (Koka and Ramdass). The article discussed an anticipated efficacy of interferon stimulated gene-15 (ISG15) inclusion with the latency-reversing agents to rejuvenate the exhausted immune responses to eliminate HIV infection in humans *in vivo*. This projection may be able to meet this inadequacy from immune exhaustion and to ensure sustained cytotoxic T lymphocyte (CTL) and natural killer (NK) cell lytic vesicles released immune responses.

The progenitor hematopoietic stem cells (HSCs) are reported to be more resilient than the mesenchymal stem cells (MSCs), when both cell types were derived from human peripheral blood and cultured *in vitro*, to examine parallels for *in vivo* feasibility and implications to therapeutic efficacy (Samundeshwari et al.). Compared to MSCs, the positive indicators for HSCs were slower growth, higher viability, and increased survival in culture conditions *in vitro*, due to the expression of telomerase, BCL2, and Notch1 genes. The differentiation potential of MSCs is curtailed due to premature apoptosis as shown by the increased expression of the apoptotic genes compared to the availability of multiple varied attributes and characteristics of the HSCs.

However, despite their above reported pre-emptive apoptotic characteristic, the human MSCs exhibited certain levels of efficacy against induced spinal cord injury (SCI) in mice (Tsuji et al.). Human amnion-derived mesenchymal stem cells (hAMSCs) when administered intravenously, were efficacious in improving mouse spinal cord gait and neuroprotective sensory function. All these physical improvements also correlated with decreased TNFα and increased bone marrow-derived neurotrophic factor (BDNF) levels, in the hAMSC-treated compared to control physiological saline treated mice. A reduced local and systemic inflammation was also observed as assayed from reduced monocytic bone marrow-derived suppressor cells (M-MDSCs) and Ly6C-positive inflammatory macrophages in the bone marrow. Hence, further evaluation of hAMSC treatment for SCI is suggested.

The above two articles published in this Research Topic may have considerations to be explored in the context of the utilization and efficacy of the HSCs and MSCs in the bone marrow (BM) microenvironment when hematopoietic stem cell transplantation (HSCT) is undertaken. The multiple effects of the chemotherapy agent busulfan dosage in the hematopoietic cell transplantation for hematological malignancies and the ensuing hematopoietic recovery have recipient age dependent consequences (Abbasizadeh et al.). In this context, the reported effects of cytotoxic conditioning with busulfan in HSCT, may have potential considerations, with MSCs in the supplementary additives. This is expected to stabilize the bone marrow (BM) microenvironment post-transplantation, although not expected to participate in the BM recovery across different age recipients.

More recent characterization and exploration of marine invertebrate stem cells (MISCs) as reviewed (Mohajer et al.), can provide a valuable opportunity to investigate their uses including in the development of therapeutic applications. Of significance, certain MISCs of species such as in starfish in regeneration of injured sites, carry a novel stem cell “spontaneous de-differentiation phenotype” that needs to be further characterized and utilized. It is discussed that such investigations and the determination, if of “orthologous” relevance, lead to the derivation of pluripotent and totipotent stem cells of the MISCs. These novel non-human counterpart stem cells may facilitate research on their transplantation into other animals in the laboratory to evaluate inter-species biological compatibility and translational efficacy, that may also include studies on epigenetic regulation post-transplantation adaptability.

The transcriptomics of gene regulation by the non-coding RNAs (ncRNAs) have multi-faceted implications in the evaluation of therapeutic candidates for pathological conditions. Non-coding exosomal RNAs of the stem cells characterized for their efficacy towards neurological disorders are reviewed (Ke et al.). The preservation of blood-brain barrier integrity by the ncRNAs encompassed in the exosomal extracellular vesicles is discussed. In this regard, a question may arise if the exosomes serve as vehicles to facilitate a “*trans*-migration” of certain specific ncRNAs of optimal “size” to “deliver” efficacy for the neurological disorders, including via engaging in signalling events.

The lingering problem of wound-healing particularly in the comorbid patients of diabetes mellitus (DM), may even prove to be lethal over a time-period for the elderly who sometimes suffer difficult to prevent injuries such as bone fractures. Investigation of the therapeutic efficacy of adipose-derived stem cells (ADSCs) to promote wound healing in DM patients employing proteomics and bioinformatics techniques, is presented (Gu et al.). It is determined that the ADSCs-induced wound-healing is enhanced through an “activation of the Wnt/β-catenin signalling pathway,” known to be involved in “cell growth, proliferation, differentiation and apoptosis.”

In conclusion, as reported in these different Research Topic articles, lie the advantages of different types of stem cell treatments with their regeneration potential to varying degrees. Continued basic research and clinical investigations of the stem cell translational applications remain a need and helped by making use of the emerging technological advancements. The stem cells such as those afflicted by the non-communicable diseases as are the cancer stem cells, or those stem cells that are targeted by the pathogens that exist in the environment, continue to require further investigational approaches in regenerative medicine.
